# Bioethical Implications in Vaccine Development, a COVID-19 Challenge

**DOI:** 10.7759/cureus.10530

**Published:** 2020-09-18

**Authors:** Sebastián Ospina Henao, Alejandro Marín Mora, Fanny Chan Solano, María L Ávila-Aguero

**Affiliations:** 1 Instituto de Investigación en Ciencias Médicas, Universidad de Ciencias Médicas, San José, CRI; 2 Centro de Estudios en Bioderecho, Ética y Salud, Universidad de Murcia, Murcia, ESP; 3 Ethics Committee, Universidad de Ciencias Médicas, San José, CRI; 4 Pediatric Infectious Diseases, Hospital Nacional De Niños Dr. Carlos Sáenz Herrera, San José, CRI; 5 Pediatric Infectious Diseases, Center for Infectious Disease Modeling and Analysis, Yale School of Public Health, New Haven, USA

**Keywords:** bioethics, clinical trial, covid-19, social value, vaccine

## Abstract

Introduction

In the last 18 years, on three occasions, coronavirus has represented a challenge for global health. Between 2002 and 2003 with Severe Acute Respiratory Syndrome, in 2012 with Middle East Respiratory Syndrome, and since the end of 2019 with severe acute respiratory syndrome coronavirus 2 (SARS-CoV-2) causing the coronavirus disease 2019 (COVID-19) pandemic, which has challenged health care models and the way of doing research, placing bioethics at the center of discussion.

Methods

On August 19, 2020, a webinar organized by the Research Institute of Medical Science (IICIMED, for its acronym in Spanish), entitled 'Bioethical Implications in Vaccine Development, a COVID-19 Challenge' took place. Three experts spoke about the importance of bioethics in the race to develop a COVID-19 vaccine, the risk involved in shortening the terms of the clinical trial phases, and how the associated risks can be minimized, in order to expedite research results.

Conclusion

With the novel SARS-CoV-2 coronavirus, critical challenges have been posed not only for public health but for research and the scientific community. A safe and effective vaccine is urgently needed to prevent COVID-19 transmission, complications, and deaths; the adherence to ethical principles required by clinical research is mandatory and closer supervision is also essential.

## Introduction

In December 2019, an acute febrile illness accompanied by severe respiratory distress syndrome was identified in Wuhan, Hubei province, China. The first cases were linked to the Hunana Seafood Market, a wet market, although unrelated cases began to emerge shortly after [1], and then the disease spread rapidly from Wuhan to all of China, with cases reported in many other countries with local transmission [2]. On January 30, 2020, the disease was declared by the World Health Organization (WHO) as a public health emergency of international concern and on March 11 it was declared a pandemic [3].

Since the first official case reported in December 2019 [1], until August 23, 2020, after almost nine months, the pandemic has caused more than 23 million infections and more than 800,000 deaths worldwide [3], as well as trillions of dollars in losses and its impact on world economies, even those of first-world developed countries. Despite public health measures aimed at preventing contagion, the pandemic has not been controlled. A vaccine is urgently needed to prevent the transmission of coronavirus disease 2019 (COVID-19) and stop the complications and deaths resulting from this disease.

According to a projection by the Imperial College COVID-19 Response Team [4], suppression, isolation, and social distancing should be maintained in some way until an effective vaccine or treatment against COVID-19 is available. In their report they explain that, in the absence of interventions, COVID-19 would have resulted in 7.0 billion infected and 40 million deaths worldwide by the end of this year. Mitigation strategies that focus on protecting the elderly and slowing down but not interrupting transmission could reduce its burden by 50%, saving 20 million lives.

## Materials and methods

On August 19, 2020, a webinar organized by the Research Institute of Medical Science (IICIMED, for its acronym in Spanish), entitled "Bioethical Implications in Vaccine Development, a COVID-19 Challenge" took place. Three experts, Dr. María Luisa Ávila Agüero, Dr. Alejandro Marín Mora, Dr. Fanny Chan Solano, and the moderator, Dr. Sebastián Ospina Henao, spoke about the importance of bioethics in the race to develop a COVID-19 vaccine, the risk involved in shortening the terms of the clinical trial phases and how the associated risks can be minimized, in order to expedite research results. This article arises from the theoretical basis of that discussion and aims to give an update on what is known so far on the subject.

## Results

In the last 18 years, on three occasions, the coronavirus has represented a challenge for global health. Between 2002 and 2003 with the Severe Acute Respiratory Syndrome (SARS-CoV), in 2012 with the Middle East Respiratory Syndrome (MERS) and now, with the new severe acute respiratory syndrome coronavirus 2 (SARS-CoV-2), critical challenges have been posed not only for public health but for research and the medical-scientific community in general [5]. As a response to these challenges, current efforts are focused on shortening the development deadlines of vaccines that demonstrate safety and efficacy in generating immunity in the population, through large, randomized and controlled trials, which turn out to be the most efficient, scientifically sound, and generalizable way to establish the efficacy of the vaccine [6,7]. However, the operational complexity inherent in a large study is compounded by the ripples of the pandemic; efficacy can be determined only if there is a match between the location of the vaccinated participants and the “hotspots” of the pandemic [8].

Vaccine development is often a lengthy process, requiring years to move from preclinical (in vitro and animal testing) to clinics and then to large-scale vaccine production and licensing. The safety of a vaccine must be confirmed by extensive animal work, followed by inoculation of dozens of humans and then increasing to thousands. The demonstration of efficacy usually depends on the collection and comparison of cases in thousands of people who are randomly given the vaccine or placebo. The speed with which COVID-19 vaccines have been developing is notably faster compared to conventional vaccines for other diseases. From the publication of the first SARS-CoV-2 sequences to phase 1, it has taken an average of six months, compared to a typical timeline that takes three to nine years [6-8].

With the accelerating pace of development of vaccines against COVID-19, by August 23, 2020 more than 135 vaccines were in preclinical trials, 21 in phase 1, 13 in phase 2, eight in phase 3, and two approved (with restriction of use and limited to certain geographical areas given the little information shared with the global scientific community on the development of these two vaccines) [9]. This is due to several factors, among them: prior knowledge of the role of spike protein in the pathogenesis of the coronavirus and evidence that neutralizing antibody against spike protein is important for immunity; the evolution of nucleic acid vaccine technology platforms that enable the creation of vaccines and the prompt manufacture of thousands of doses once a genetic sequence is known; and developmental activities that can be performed in parallel, rather than sequentially, without increasing risks to study participants [8]. Compressing and overlapping the clinical phases of vaccine development, accelerating the transition between them, driving efficacy studies to produce results in a short period of time, and pursuing large-scale vaccine manufacturing before regulatory approval results in a bioethical dilemma that must be addressed within the context of the outbreak [6,10].

Among the proposed models, one of the most controversial, due to the ethical considerations it raises, is the Controlled Human Infection Model (CHIM) as an alternative to accelerate the development of the COVID-19 vaccine as much as possible. This model is based on the fact that the participants are exposed to the pathogen in a natural environment and can include heterogeneous populations with higher risk of contracting the disease or serious results. Due to its design, the controlled nature of CHIM limits the possibility of generalizing to predict the effectiveness of a candidate vaccine against natural exposure. However, in order to justify this model, the first step is to demonstrate its high social value, since it generates not only great uncertainty but also controversy [6,11].

CHIM ethics are little explored, and the COVID-19 ethical processes have largely focused on whether the risks are acceptable and participants could give high-quality informed consent, using rigorous procedures to maximize understanding of the participants. In this informed consent process, the individual rights of the participants cannot be violated under the guise of an emergency response, but special attention must be paid to both individual rights and the global public health emergency [6,10,11].

## Discussion

The COVID-19 pandemic is a stark reminder of the ongoing challenge that emerging and re-emerging infectious pathogens create for the need for constant surveillance, rapid diagnosis, and robust research. Understanding the basic biology of new organisms requires an international multisectoral program, focused above all on a solid public health response that, during a pandemic, must adhere to fundamental ethical and scientific principles. This will help to quickly and definitively determine the safety and efficacy of interventions and thus provide rapid access to the most effective therapies for the largest number of patients [5,12].

The fundamental ethical and scientific principles for conducting trials in an emergency context such as the COVID-19 pandemic are: ethical conduct to avoid exploitation, partnership with researchers and officials from affected countries, scientific validity, independent review, scientific oversight, and transparency. Associations between these principles are particularly important in outbreak situations, and local realities can complicate the development of these relationships. First, in the case of government-to-government partnerships, a diplomatic invitation is an extremely useful first step. Second, the country's capacity to conduct clinical research must be carefully evaluated. Third, regulatory oversight may need to be strengthened, as while building strong local partnerships and local capacity is a resource-intensive and time-consuming process, it establishes the critical infrastructure for conducting testing to the highest ethical and scientific standards to increase its probability of success and reduce the possibility of inadvertent harm to study populations in the context of the current outbreak and in the future [12].

The WHO issued a draft of an "R & D Blueprint" [13] entitled "Feasibility, Potential Value and Limitations of Establishing a Closely Monitored Challenge Model of Experimental COVID-19 Infection and Illness in Healthy Young Adult Volunteers", where they start from the basis of the role that CHIMs have had in the development of certain vaccines, and they explain the importance of being able to guarantee the conditions to carry out these studies safely and the urgency in defining which should be the priority objectives for such studies, since clinical trials in outbreak situations should also ensure that the participants to be studied have a plausible benefit and that the trial designs used are scientifically sound and justified [12].

## Conclusions

With the novel SARS-CoV-2 coronavirus, critical challenges have been posed not only for public health but for research and the scientific community at large. This is why a safe and effective vaccine is urgently needed to prevent the spread of COVID-19 and thus stop the complications and deaths resulting from the transmission of the disease, being now more than ever mandatory to adhere to standard ethical principles that clinical research requires and even stricter control and supervision measures.

The role that the Ethics Committees (EC) and Institutional Review Boards (IRB) are playing is key. The responsibility of evaluating and reviewing a clinical research protocol does not only remain with the traditional review. Faced with a pandemic scenario associated with scientific research, the ECs and the IRBs must supervise very close the conduct of the study and a constant assessment should be established to guarantee a vigilance of the participants' safety and that the processes are carried out in the most rigorous manner.

Vulnerable populations become even more vulnerable under emergency conditions, and the possibility of influencing the decision of potential participants is just around the corner due to the way in which the general public receives information.
